# Sex Differences in the Incidence and Outcomes of Patients Hospitalized by Idiopathic Pulmonary Fibrosis (IPF) in Spain from 2016 to 2019

**DOI:** 10.3390/jcm10163474

**Published:** 2021-08-06

**Authors:** Belén López-Muñiz Ballesteros, Marta López-Herranz, Ana Lopez-de-Andrés, Valentín Hernandez-Barrera, Rodrigo Jiménez-García, David Carabantes-Alarcon, Isabel Jiménez-Trujillo, Javier de Miguel-Diez

**Affiliations:** 1 Respiratory Department, Hospital Universitario Infanta Leonor, 28031 Madrid, Spain; belenlmb@yahoo.es; 2 Nursing Department, Faculty of Nursing, Physiotherapy and Podology, Universidad Complutense de Madrid, 28040 Madrid, Spain; 3 Department of Public Health & Maternal and Child Health, Faculty of Medicine, Universidad Complutense de Madrid, 28040 Madrid, Spain; anailo04@ucm.es (A.L.-d.-A.); rodrijim@ucm.es (R.J.-G.); dcaraban@ucm.es (D.C.-A.); 4 Preventive Medicine and Public Health Teaching and Research Unit, Health Sciences Faculty, Universidad Rey Juan Carlos, Alcorcón, 28922 Madrid, Spain; valentin.hernandez@urjc.es (V.H.-B.); isabel.jimenez@urjc.es (I.J.-T.); 5 Respiratory Department, Hospital General Universitario Gregorio Marañón, Instituto de Investigación Sanitaria Gregorio Marañón (IiSGM), 28007 Madrid, Spain; javier.miguel@salud.madrid.org; 6 Department of Medicine, Faculty of Medicine, Universidad Complutense de Madrid, 28040 Madrid, Spain

**Keywords:** idiopathic pulmonary fibrosis, incidence, in-hospital mortality, sex, Spain

## Abstract

(1) Background: To assess sex differences in the incidence, characteristics, procedures and outcomes of patients admitted with idiopathic pulmonary fibrosis (IPF); and to analyze variables associated with in-hospital mortality (IHM). (2) Methods: We analyzed data collected by the Spanish National Hospital Discharge Database, 2016–2019. (3) Results: We identified 13,278 hospital discharges (66.4% men) of IPF (primary diagnosis 32.33%; secondary diagnosis: 67.67%). Regardless of the diagnosis position, IPF incidence was higher among men than women, increasing with age. Men had 2.74 times higher IPF incidence than women. Comorbidity was higher for men in either primary or secondary diagnosis. After matching, men had higher prevalence of pulmonary embolism and pneumonia, and women of congestive heart failure, dementia, rheumatoid disease and pulmonary hypertension. Invasive ventilation, bronchoscopy and lung transplantation were received more often by men than women. IHM was higher among men with IPF as primary diagnosis than among women and increased with age in both sexes and among those who suffered cancer, pneumonia or required mechanical ventilation. (4) Conclusions: Incidence of IPF was higher among men than women, as well as comorbidity and use of bronchoscopy, ventilation and lung transplantation. IHM was worse among men than women with IPF as primary diagnosis, increasing with age, cancer, pneumonia or mechanical ventilation use.

## 1. Introduction

Idiopathic pulmonary fibrosis (IPF) is a fibrosing interstitial pneumonia of unknown cause, which usually affects elderly adults and is characterized by a radiological or pathological pattern of usual interstitial pneumonia [1]. The diagnosis is made, after excluding other causes of interstitial lung disease, with the combination of the radiological and pathological pattern, in those patients who have undergone a surgical biopsy, and the subsequent discussion by a multidisciplinary team [2,3].

IPF is a disease with a poor prognosis, with a mean survival of 2–5 years, which can vary from 27.4 months in severe forms (FVC <50%) to 55.6 months in mild forms (FVC >70%). The appearance of anti-fibrotic drugs has improved life expectancy, reaching an average of 8 years actually [4]. The course of the disease is variable, with a difficult to predict prognosis, with respiratory failure related to disease progression being the main cause of mortality [5].

IPF not only has a severe prognosis, but also it is the most common idiopathic interstitial pneumonia. Although its incidence has been increasing over the years, it is difficult to estimate it accurately because the studies carried out to date have used different methodologies and vary depending on the race and the geographical area studied. Thus, incidence figures have been described in the literature from 0.2 per 100,000 to 93.7 per 100,000 inhabitants, with values of 3 to 9 per 100,000 inhabitants per year in Europe and North America. In Spain, it is estimated that the incidence is 13 per 100,000 inhabitants in women and 20 per 100,000 inhabitants in men, with an approximate prevalence of 8000–12,000 patients [6,7,8,9]. Variable mortality figures have also been described in different countries. Thus, in the European WHO registry, in which patients with IPF from 17 countries were collected between the years 2001 to 2013, a mortality of 3.75 per 100,000 in men and 1.50 per 100,000 in women was estimated. In addition, this study found variations in the mortality figures over time, so that they tended to decrease in countries such as Austria or Croatia, while in others such as the United Kingdom, Finland or Portugal, an increasing trend in mortality has been reported in recent years [10].

Hospital records are crucial to knowing the incidence of IPF, the characteristics of the disease, its evolution over time, as well as its behavior before and after hospitalization [11,12]. In the study by Farrand et al., in which 691 patients with IPF were analyzed compared to 3452 controls, a greater consumption of healthcare resources was detected in patients with IPF since the year before diagnosis compared to the control group [11]. 

The objectives of the study were the following: (1) to assess sex-differences in the incidence, clinical characteristics, diagnostic and therapeutic procedures and in-hospital outcomes of patients admitted with a diagnosis, in primary or secondary position, of idiopathic pulmonary fibrosis (IPF) in Spain from 2016 to 2019; (2) to analyze the variables associated with in-hospital mortality (IHM) for men and women hospitalized with IPF as primary diagnosis.

## 2. Materials and Methods

### 2.1. Study Design and Data Source

We carried out an epidemiological cohort study using data provided by the Spanish National Hospital Discharge Database (SNHDD). The SNHDD is a mandatory registry that includes data on all the hospital admissions in any Spanish hospital, public and private. The database includes basic demographic data (age, sex, province of residence), length of hospital stays (LOHS), up to a maximum of 20 diagnoses and procedures (diagnostic and therapeutic) conducted during the admission and the outcome of the hospitalization. The SNHDD is fulfilled using the information included in the patient’s discharge report and in the hospital procedures databases. Since 2016, the SNHDD has been using the International Classification of Disease version 10 (ICD-10) to codify diagnosis and procedures. More details of the SNHDD are provided elsewhere [13].

### 2.2. Study Population

We analysed data from adults (aged 18 years or over) collected by the SNHDD in 2016, 2017, 2018 and 2019. 

Our study population includes all patients discharged with a diagnosis of IPF in any diagnostic position of the SNHDD (ICD-10 code J84.112).

The analysis was conducted separately for women and men, to assess sex differences, according to study variables. The study population was also analysed according to the position of the IPF diagnosis in the discharge report (primary or secondary). The primary diagnosis is the clinical conditions that, in the opinion of the physician who completes the discharge report, is the principal cause for admitting the patient to the hospital. The secondary diagnosis are other conditions present at admission, or diagnosed during the hospitalization, that are not the main reason for the hospital admission, but have affected the duration, procedures, or outcome of the hospitalization. Therefore, it is logical to think that only in patients with IPF as a primary diagnosis this condition was the cause of death. For those with IPF in a secondary position the cause of death would, in most cases, not be directly related to IPF but with other condition that resulted in the hospitalization.

Subjects with missing values or with “unknown” or “undetermined” in their variables of sex, age, LOHS, or outcome of the hospitalization were excluded from the study population.

### 2.3. Study Variables

Our main study variables were the incidence rates and the IHM after IPF hospitalization according to diagnosis position and sex.

To estimate incidences, we used population data provided by the Spanish National Statistics Institute [14]. The incidences are calculated per 100,000 inhabitants. 

The remaining study variables included age, conditions in the Charlson Comorbidity Index (CCI), pulmonary embolism, pulmonary hypertension and pneumonia. The codes for these variables, located in any diagnosis fields of the database are shown in Table S1. The algorisms and codes to identify the conditions included in the CCI are those described by Sundararajan V et al. [15].

We retrieved data about tobacco use, therapeutic and diagnosis procedures such as oxygen prior to hospitalization, receipt of mechanical ventilation (invasive and non-invasive), computed tomography of the chest, respiratory function tests, bronchoscopy, lung scintigraphy and lung transplant (see Table S1 for ICD10 codes).

### 2.4. Propensity Score Matching

Propensity score matching (PSM) was used to make the baseline characteristic of the study sub-groups, namely men and women and of those with and without IPF as a primary diagnosis, more comparable [16]. As described before, to conduct PSM we used multivariable logistic regression we obtained a propensity score (PS) for each patient that was then matched with a patient in the subgroup to be compared with a similar PS value [17]. The variables included to obtain the PS were age and the chronic conditions present at admission.

### 2.5. Statistical Analysis

We presented our results for men and women separately and stratified by the diagnosis position of IPF.

Poisson regression models were used to assess differences in the incidences, according to sex and diagnosis position for the period 2016–2019.

Descriptive analysis included means or medians with standard deviations (SD) or interquartile ranges (IQR), as required, for continuous variables and absolute frequencies and percentages for categorical variables.

Bivariate analysis included *t*-test, Mann–Whitney test or Fisher exact test, depending on the types of variables being compared. 

Multivariate logistic regression has been used to identify which variables were associated with IHM among men and women with IPF codified in primary diagnosis position. The construction of these models was undertaken following the recommendation of Hosmer et al. [17].

Stata version 14 program (Stata, College Station, TX, USA) was used as statistical software. 

### 2.6. Sensitivity Analysis

It has been reported that when patients with interstitial lung disease such as IPF have any rheumatoid disease, this lung disease should be diagnosed as not “idiopathic” but connective tissue disease-associated (secondary) in most cases [18]. In our investigation, we decided to use the ICD 10 code J84.112 to identify IPF, beside whether a code for any rheumatoid diseases was present so that these cases may be misclassified. In order to control this bias, we have analyzed the database excluding those patients hospitalized with IPF as primary or secondary diagnosis with any code for rheumatoid disease in any diagnosis position.

### 2.7. Ethical Aspects

The anonymized databases of the SNHDD are provided free of charge by the Spanish Ministry of Health after a justified request form is fulfilled and sent [19]. Therefore, the need to obtain the approval by an ethics committee is waived according to the Spanish legislation.

## 3. Results

We identified 13,278 hospital discharges (66.4% men and 33.56% women) of patients aged ≥18 years admitted with a primary diagnosis of IPF (4294; 32.33%) or secondary diagnosis of IPF (8984; 67.67%) in Spain between 2016 and 2019.

Regardless of the diagnosis position, the overall incidence of IPF per 100,000 inhabitants was higher among men than women (11.85 per 100,000 men vs. 5.64 per 100,000 women; *p* < 0.001). The result of the Poisson regression adjusted by age showed that men had 2.74 (IRR 2.74, 95% CI 2.64–2.84) times higher incidence of IPF than women.

When Poisson regression analysis was conducted to compare differences in the incidences between men and women, for primary and secondary diagnosis of IPF, we obtained an IRR of 3.06 (95% CI 2.64–2.84) and IRR 2.61 (95% CI 2.50–2.73), respectively.

When the number of cases per year were compared, we observed that the proportion of primary and secondary diagnosis seems to increase among men and decrease among women.

For both sexes, overall incidence increased with age, reaching the highest values in the ≥80 years’ group in women (51.47 per 100,000 women) and in the 70–79 years’ group in men (35.16 per 100,000 men). In all age strata men had significantly higher figures than women (Table 1). 

As can be seen in Table 2, women hospitalized with IPF as primary diagnosis (74.7 years vs. 71.69 years; *p* < 0.001) or secondary diagnosis (78.91 years vs. 76.06 years; *p* < 0.001) were significantly older than men. On the other hand, the mean values of CCI were higher for men in either primary (1.15 vs. 0.94; *p* < 0.001) or secondary diagnosis (2.15 vs. 1.67; *p* < 0.001). 

The most frequent chronic conditions found in the discharge reports of men with a diagnosis of IPF were chronic pulmonary disease (27.9%), diabetes (25.97%) and congestive heart failure (20.55%). Among women, congestive heart failure (25.86%), diabetes (21.84%) and chronic pulmonary disease (20.36%) were the most prevalent conditions. 

Beside the diagnosis position of IPF, in men the following conditions were more frequently codified than women: myocardial infarction, peripheral vascular disease, chronic pulmonary disease, and diabetes. Women had more dementia and rheumatoid disease.

Overall, pulmonary hypertension was found in (13.54% of women and 11.28% of men *p* < 0.001), with similar figures beside the IPF diagnosis position.

Pneumonia was an uncommon diagnosis when IPF was the primary diagnosis, 3% for men and 3.7% for women (*p* = 0.231) but appeared more frequently if IPF was in a secondary position (13.84% among men and 12.03% among women; *p* = 0.016).

Tobacco use was much more prevalent among men than women regardless of whether IPF was codified in a primary or secondary position with figures around 50% for men and 10% for women. 

Regarding procedures, oxygen prior to hospitalization, was being used by 27.5% of men and 29.41% of women (*p* = 0.021). Invasive ventilation and bronchoscopy were received by more men than women beside the diagnosis position. Lung transplant was conducted in 162 men and 31 women with IPF (1.84% vs. 0.7%; *p* < 0.001), in most cases with IPF as a primary diagnosis.

The IHM observed for the period 2016–2019 were 17.98% for men and 14.12% for women with IPF as primary diagnosis (*p* = 0.002) and 15.69% and 13.33% (*p* = 0.003) respectively, when it was codified as secondary diagnosis.

The distribution of clinical conditions and procedures after PSM are shown in Table 3. As can be seen after matching, the differences in the prevalence of most clinical conditions became not significant. However, for the entire study population men still had higher prevalence of pulmonary embolism and pneumonia and women of congestive heart failure, dementia and rheumatoid disease and pulmonary hypertension.

The proportion of men who received invasive ventilation, bronchoscopy and lung transplant remained significantly higher than women after PSM. 

The IHM after PSM was higher among men than women with IPF as primary (18.47% vs. 14.12%; *p* = 0.003) and secondary diagnosis (16.36% vs. 13.33%; *p* = 0.001).

The results of the multivariable analysis to identify which variables are associated with dying in the hospital after IPF as primary diagnosis, according to sex, are shown in Table 4. In both sexes the IHM increased with age and was higher among those who suffered cancer, pneumonia or required mechanical ventilation (invasive or not invasive). The need of oxygen prior to hospitalization was also a risk factor for men and women with IPF as primary diagnosis.

Bronchoscopy and lung transplant were procedures associated to a better survival among men with IPF as primary diagnosis.

When both men and women were analysed together to assess the effect of sex on the IHM for patients with IPF as primary diagnosis we obtained the results shown in Supplementary Table S2. This multivariable logistic regression confirms the results of the PSM showing that men have higher IHM than women in those discharged with IPF as primary diagnosis (OR 1.43 95% CI 1.15–1.77).

The protective effect of diabetes in the IHM among those with IPF (OR 0.66; 95% CI 0.48–0.9) is remarkable.

### Sensitivity Analysis

Table S3 shows the incidence and in-hospital mortality of hospital admissions with IPF, excluding all patients with a diagnosis code for any rheumatoid disease in Spain from 2016 to 2019, according to diagnosis position, sex, year and age groups. As can be seen in this table, the incidence, IHM and distribution of the study population were very similar to the results shown in Table 1. The incidence and the IHM of IPF as a primary or secondary diagnosis, when all patients with any code for rheumatoid disease were excluded, remained significantly higher among men than women. Furthermore, according to the total number of cases from 2016 to 2019, an increment is observed among men and a reduction among women. This is the same time trend observed with the entire study population (Table 1).

The results of the logistic regression to identify variables associated with IHM among men and women with a primary diagnosis of IPF are shown in Table S4. The variables found to be significantly associated were the same ones found before patients with rheumatoid disease were excluded.

## 4. Discussion

In our study, the incidence of IPF in men was almost three times higher than that of women, with an age-adjusted incidence of 2.74. According to our results, the differences in PFI between male and female patients could be associated, at least in part, with a lower level of smoking in women compared to men, since cigarette smoking is one of the most recognized risk factors for development of IPF [20]. The lower rate of CT performed in women could also influence these results, since sex-differences have previously been described in the diagnosis or treatment of respiratory diseases [21]. In this sense, data on antifibrotic therapy were not available for our study, but the effect of antifibrotic therapy on pulmonary function does not seem to differ between men and women [22].

Our results are similar to those of other series [23,24,25], having shown that the incidence increases after 65 years of age [3]. In our population the mean age of women was higher than among men. However, other authors such as Raghu et al. have not found significant differences with regard to age and sex [12,24,26].

Comorbidities in IPF worsen prognosis and delay diagnosis. Its frequency, like the disease itself, increases with increasing age. In the study by Farrand et al. [11] the Charlson index was significantly higher in patients with IPF compared to the control group (3.2 ± 2.5 vs. 2.0 ± 2.2). In our study, comorbidities were more frequent in men, the most common being chronic obstructive pulmonary disease (COPD), diabetes mellitus, and heart failure. These results differ from the Spanish National SEPAR registry, where the main associated comorbidity was gastroesophageal reflux (12.8%), followed by emphysema (12.1%), coronary disease (8.6%) and arterial hypertension (6.2%) [27].

The most frequent comorbidity in our study was COPD. This could be justified because tobacco is a risk factor for developing the two entities, both of which can coexist in the combined fibrosis-emphysema syndrome, described by Professor Cottin in 2005 [28,29]. The cause of the association between both diseases is unknown and is not fully explained by the association with tobacco. In animal models, the surfactant protein D, TNF-α, inteleukin-8 (IL-8) and elastase, have been shown to have a potential pathogenic component, although these data have not been confirmed to date in humans [30]. The prevalence of the association of fibrosis and emphysema is unknown and varies in different studies [31,32,33]. Thus, the prevalence in our series was 27.9%, while in that of Ryerson et al. [31], in which 365 patients with IPF were analyzed, it was 8%, and in that of Otsuka et al. [32], in which 831 patients who underwent resection for lung cancer were analyzed, it was 2.8%. However, in the series by Ye et al. [33], in which 125 patients with IPF were followed for 48 months, a prevalence of fibro-emphysema of 56% was detected. These differences are probably due to the different methodology used in the studies and to the fact that sometimes the criteria for the definition of fibro-emphysema and its radiological quantification are not clear.

Diabetes mellitus was the second most frequent comorbidity in our series in both men and women. In the study of Enomoto et al. [34], the prevalence of diabetes in patients with IPF was 32%. This study suggests that diabetes is a risk factor for developing IPF. Although the cause is unknown, hyperglycemia could act directly on alveolar cell damage or by participating in the activation of other pro-inflammatory or pro-fibrotic mediators [35].

Multiple studies have shown a high prevalence of cardiovascular disease in patients with IPF, this comorbidity being more frequent in it than in other interstitial diseases. In our series, it was associated with 20.55% of men with IPF and 25.86% of women. In the sample of Hubbard et al. [36], the prevalence of cardiovascular, cerebrovascular and thromboembolic diseases was analyzed in a cohort of 920 patients with IPF and 3593 controls, demonstrating an increased risk of acute coronary disease and deep vein thrombosis in patients previously diagnosed with IPF. Nathan et al. [37] described a 65.8% prevalence of coronary artery disease in a series of 73 patients with IPF who underwent catheterization prior to transplantation. The cause of the association between IPF and cardiovascular events is unknown, although it has been suggested that the severe hypoxemia caused by the disease could contribute to developing these events. It is also unknown whether this association is due to IPF or the age of the patients and its association with other risk factors such as smoking or diabetes. What is clear is that the appearance of cardiovascular disease in patients with IPF makes it difficult to manage this disease and worsens its prognosis [30].

In our study, more interventions were performed in men, who underwent fiberoptic bronchoscopy more frequently than women and also received treatment with non-invasive mechanical ventilation more frequently. One of the main causes of hospital admission is acute respiratory failure, which may require the use of mechanical ventilation. In the study by Alqalyoobi et al. [38], patients admitted with respiratory failure had a mortality of 20.5%, this figure increasing to 40.8% in those who required non-invasive mechanical ventilation. However, mortality was lower in patients who were admitted to referral hospitals compared to those who were admitted to regional hospitals. Hence the importance of establishing early diagnosis and treatment protocols in these patients. Transplantation was also more frequent in men than in women, which could be explained by the higher prevalence of the disease in males and their younger age at the time of diagnosis. 

In our investigation, among those with IPF as a primary diagnosis, after multivariable analysis the variables associated analysis with IHM in both sexes included aging, pneumonia and cancer. There are different explanations to justify this finding. It could be due to the fact that, in elderly people, the disease is more aggressive due to the lower response capacity of the organism, the diagnostic delay is more frequent due to the non-specificity of the clinic and it is more frequent to adopt a conservative attitude, performing fewer diagnostic tests. In addition, older people tend to have a greater number of comorbidities, which by themselves worsen the prognosis of the disease. To this must be added a worse tolerance to anti-fibrotic treatment in this group, with a greater number of side effects [39]. 

We found that suffering cancer was associated to a higher risk of dying during the hospitalization among men and women with a primary diagnosis of IPF. Different studies have described an increase in the incidence of cancer in patients with IPF, although the exact prevalence is unknown today. Ozawa et al. [40] analyzed a series of 103 patients with IPF, of whom 20.4% developed lung cancer during the 10-year follow-up of the disease. In the series by Tomasetti et al. [41], poorer survival was demonstrated in IPF patients who developed lung cancer, although mortality in this series was mainly due to lung cancer progression and not IPF [39].

The use of anti-fibrotic drugs has contributed to improving the prognosis of IPF, with an increase in the time free of its progression and a tendency to decrease mortality, although there have been contradictory results in different studies. Thus, for example, in the American series by Jeganathan et al. [42], in which patients with IPF were analyzed from 2004 to 2017, a decrease in mortality adjusted by age and sex was detected, being 4.1% in men (from 75.4/10,000,000 inhabitants to 72.4%/1,000,000 inhabitants in 2017) and 13.4% in women (46.3/10,000,000 inhabitants in 2004 to 40.1/1,000,000 inhabitants in 2017), the main cause of death being the evolution of the disease itself. The only age group where an increase in mortality was detected was those over 85 years of age. However, this trend could be due, in addition to anti-fibrotic treatment, to better knowledge and management of the disease and to a reduction in tobacco use. In any case, the results of this series contradict those obtained in other older studies, such as that of Olson et al. [43], who observed that mortality increased between 1992–2003.

On the other hand, fiberoptic bronchoscopy, lung transplantation, or the presence of diabetes in our study were associated with better survival. The protective effect of fiberoptic bronchoscopy on IMH may be due to the fact that most patients undergoing bronchoscopy were admitted to the hospitals for diagnostic evaluation at the early and stable stage; in other words, fewer patients underwent bronchoscopy in the setting of serious acute events such as acute exacerbation and at the end-stage of IPF. On the other hand, the protective effect of lung transplantation on IMH can be explained by the fact that most patients undergoing lung transplantation could be successfully discharged with recovery. Lung transplantation is the only treatment in advanced stages of the disease, achieving significant functional improvement and improved survival. In fact, survival figures at one year of 81%, 64% at 3 years and 51% at 5 years have been described, this being similar to that of single or double lung transplantation [44]. 

In our series, the presence of diabetes was a protective factor for mortality. Nevertheless, this finding should be viewed with caution, especially when retrospective data are assessed. It could be related to the phenomenon of paradoxical obesity. In this way, in people with diabetes, overweight has been associated with a lower risk for in-hospital mortality [45]. It is also possible that patients with diabetes are admitted to the hospital more frequently than patients without diabetes with the same clinical severity. This would lead to a selection bias that could partly explain the lower IHM among patients with diabetes. However, in other series such as Hyldgaard et al. [46], patients with IPF and diabetes had a shorter survival. This increase in mortality was not related to the use of antidiabetics or intravenous corticosteroids, but the prognosis was worse in those patients in whom the diagnosis of diabetes was prior to that of IPF. 

Our study has several limitations. The main one is that the data were obtained from an administrative database, in which the pathologies and procedures were coded according to the ICD-10-CM. Given this, we cannot know who made the diagnosis of IPF, a pulmonologist or other specialists, or if the diagnosis was confirmed in a multidisciplinary discussion or was established by a group of experts. On the other hand, anonymity precludes the extraction of specific data (i.e., people who moved from one hospital to another could appear twice). 

Previous authors have used ICD10 and ICD 9 codes in administrative databases to assess the epidemiology of IPF in Canada [23], Italy [47], Sweden [48], Japan [49] and USA [24], among others. Ley at al [50] using the Kaiser Permanente Northern California database estimated that the positive predictive value of the IPF algorithm based on ICD9 was only 42.2% (95% CI, 30.6 to 54.6%) with a sensitivity of 55.6% (95% CI, 21.2 to 86.3%). Hopkins et al. [23] used ICD10 codes to investigate the prevalence and incidence of IPF in Canada with an administrative database suggesting that this disease classification may be more specific than ICD-9 codes. The reason for the higher validity of ICD10 is that the term IPF was not included in the ICD-9 codes, and ICD-9 codes may classify more patients with other common ILDs such as diffuse pulmonary fibrosis [23]. As far as we know, no validation studies of the ICD10 IPF code have been carried out so far. This can be explained by the low prevalence of this disease, which makes it very difficult to review the medical records of a few and very geographically dispersed patients.

Another limitation of studies which only use hospital admission databases is that they cannot detect those patients who are followed solely through outpatient visits and who are never hospitalized (e.g., those who die before their first hospitalization) [51].

However, as highlighted in a review of epidemiological studies in IPF, the analysis of health care databases is the most common methodologies used to identify cases of IPF. This approach provides information from a large population without the expenditure required by the creation of a national registry; moreover, it is critical for accruing a sufficient sample size for epidemiological studies for rare diseases such as IPF [51]. These authors conclude that despite the aforementioned limitations, health care administrative databases are useful tools to investigate the epidemiology of a rare disease like IPF [51]. 

The SNHDD does not collect the specific cause of death, so it is possible that some patients with a primary diagnosis of IPF died as a consequence of other conditions.

In our investigation, we considered that all hospitalizations with an ICD 10 code for IPF (J84.112) referred to a patient who suffered this disease beside the presence of a code for any rheumatoid diseases. This may result in an overestimation of the real incidence of IPF as patients with rheumatoid diseases may have fibrosis secondary to these diseases. In any case, the results of the sensitivity analyses, excluding patients with any rheumatoid diseases, are very similar, suggesting that the effect of potential misclassification is unlikely to have significantly altered the major findings of this study, and would not affect the sex differences found in our study. However, future studies with more detailed clinical data are required to confirm our conclusions. 

Despite these limitations, the SNHDD has the advantage of being part of the Spanish National Health System, which covers almost 100% of hospital admissions in Spain [18]. In addition, Spain is a large country and has a public health system that provides complete and free medical coverage to the entire population. Thus, patients come from a wide variety of socioeconomic categories, which contributes to improving the external validity of our results. 

## 5. Conclusions

In conclusion, this study provides solid data on the sociodemographic characteristics of patients admitted for IPF, their associated comorbidities, and the main causes of mortality. All of this offers us a better understanding of the disease, something that can be useful for developing new management strategies to deal with it.

## Figures and Tables

**Table 1:** Incidence of hospital admissions with idiopathic pulmonary fibrosis (IPF) in Spain from 2016 to 2019 according to diagnosis position, sex, year and age groups.

	Primary			Secondary			Both		
	Men	Women	*p*-Value	Men	Women	*p*-Value	Men	Women	*p*-Value
Incidence (2016–2019), *n* (Inc/10^5^)	2998 (4.03)	1296 (1.64)	<0.001	5825 (7.82)	3159 (4)	<0.001	8823 (11.85)	4455 (5.64)	<0.001
2016, *n* (%)	714 (23.82)	361 (27.85)	0.005	1329 (22.82)	828 (26.21)	<0.001	2043 (23.16)	1189 (26.69)	<0.001
2017, *n* (%)	811 (27.05)	372 (28.7)	0.266	1601 (27.48)	937 (29.66)	0.028	2412 (27.34)	1309 (29.38)	0.013
2018, *n* (%)	681 (22.72)	282 (21.76)	0.490	1454 (24.96)	678 (21.46)	<0.001	2135 (24.2)	960 (21.55)	0.001
2019, *n* (%)	792 (26.42)	281 (21.68)	0.001	1441 (24.74)	716 (22.67)	0.028	2233 (25.31)	997 (22.38)	<0.001
Age 18–59 years, *n* (%)	355 (11.84)	122 (9.41)	0.020	370 (6.35)	169 (5.35)	0.056	725 (8.22)	291 (6.53)	0.001
Age 60–69 years, *n* (%)	835 (27.85)	265 (20.45)	<0.001	993 (17.05)	336 (10.64)	<0.001	1828 (20.72)	601 (13.49)	<0.001
Age 70–79 years, *n* (%)	1111 (37.06)	388 (29.94)	<0.001	2057 (35.31)	882 (27.92)	<0.001	3168 (35.91)	1270 (28.51)	<0.001
Age 80 year or over, *n* (%)	697 (23.25)	521 (40.2)	<0.001	2405 (41.29)	1772 (56.09)	<0.001	3102 (35.16)	2293 (51.47)	<0.001

Inc/10^5^: Incidence per 100,000 people. *p* value for comparison of men versus women.

**Table 2:** Prevalence of clinical conditions, procedures and in-hospital outcomes in patients hospitalized with idiopathic pulmonary fibrosis (IPF) in Spain, according to sex and diagnosis position.

	Primary			Secondary			Both		
	Men	Women	*p*-Value	Men	Women	*p*-Value	Men	Women	*p*-Value
Age, mean (SD)	71.69 (10.4)	74.7 (11.22)	<0.001	76.06 (10.21)	78.91 (10.44)	<0.001	74.58 (10.48)	77.68 (10.84)	<0.001
CCI, mean (SD)	1.15 (1.38)	0.94 (0.92)	<0.001	2.15 (2)	1.67 (1.57)	<0.001	1.81 (1.77)	1.46 (1.41)	<0.001
Myocardial infarction, *n* (%)	203 (6.77)	9 (0.69)	<0.001	528 (9.06)	135 (4.27)	<0.001	731 (8.29)	144 (3.23)	<0.001
Congestive heart failure, *n* (%)	434 (14.48)	190 (14.66)	0.875	1379 (23.67)	962 (30.45)	<0.001	1813 (20.55)	1152 (25.86)	<0.001
Peripheral vascular disease, *n* (%)	163 (5.44)	11 (0.85)	<0.001	528 (9.06)	94 (2.98)	<0.001	691 (7.83)	105 (2.36)	<0.001
Cerebrovascular disease, *n* (%)	62 (2.07)	33 (2.55)	0.328	321 (5.51)	191 (6.05)	0.296	383 (4.34)	224 (5.03)	0.073
Dementia, *n* (%)	38 (1.27)	32 (2.47)	0.004	205 (3.52)	217 (6.87)	<0.001	243 (2.75)	249 (5.59)	<0.001
Chronic pulmonary disease, *n* (%)	653 (21.78)	237 (18.29)	0.010	1809 (31.06)	670 (21.21)	<0.001	2462 (27.9)	907 (20.36)	<0.001
Rheumatoid disease, *n* (%)	51 (1.7)	50 (3.86)	<0.001	187 (3.21)	261 (8.26)	<0.001	238 (2.7)	311 (6.98)	<0.001
Peptic ulcer disease, *n* (%)	18 (0.6)	5 (0.39)	0.376	35 (0.6)	13 (0.41)	0.240	53 (0.6)	18 (0.4)	0.142
Mild liver disease, *n* (%)	148 (4.94)	54 (4.17)	0.274	297 (5.1)	123 (3.89)	0.010	445 (5.04)	177 (3.97)	0.006
Diabetes, *n* (%)	693 (23.12)	242 (18.67)	0.001	1598 (27.43)	731 (23.14)	<0.001	2291 (25.97)	973 (21.84)	<0.001
Diabetes with complications, *n* (%)	34 (1.13)	15 (1.16)	0.947	184 (3.16)	66 (2.09)	0.003	218 (2.47)	81 (1.82)	0.017
Hemiplegia or paraplegia, *n* (%)	5 (0.17)	1 (0.08)	0.470	29 (0.5)	22 (0.7)	0.232	34 (0.39)	23 (0.52)	0.276
Renal disease, *n* (%)	240 (8.01)	111 (8.56)	0.539	1027 (17.63)	550 (17.41)	0.793	1267 (14.36)	661 (14.84)	0.461
Cancer, *n* (%)	83 (2.77)	21 (1.62)	0.025	525 (9.01)	125 (3.96)	<0.001	608 (6.89)	146 (3.28)	<0.001
Metastatic solid tumor, *n* (%)	31 (1.03)	6 (0.46)	0.063	285 (4.89)	44 (1.39)	<0.001	316 (3.58)	50 (1.12)	<0.001
Moderate or severe liver disease, *n* (%)	17 (0.57)	9 (0.69)	0.621	114 (1.96)	26 (0.82)	<0.001	131 (1.48)	35 (0.79)	0.001
AIDS/HIV, *n* (%)	2 (0.07)	0 (0)	0.352	9 (0.15)	1 (0.03)	0.095	11 (0.12)	1 (0.02)	0.064
Pulmonary embolism, *n* (%)	34 (1.13)	10 (0.77)	0.279	114 (1.96)	43 (1.36)	0.040	148 (1.68)	53 (1.19)	0.030
Pulmonary hypertension, *n* (%)	394 (13.14)	179 (13.81)	0.554	601 (10.32)	424 (13.42)	<0.001	995 (11.28)	603 (13.54)	<0.001
Pneumonia, *n* (%)	90 (3)	48 (3.7)	0.231	806 (13.84)	380 (12.03)	0.016	896 (10.16)	428 (9.61)	0.320
Tobacco use, *n*%	1546 (51.57)	151 (11.65)	<0.001	2829 (48.57)	288 (9.12)	<0.001	4375 (49.59)	439 (9.85)	<0.001
Oxygen prior to hospitalization, *n* (%)	929 (30.99)	394 (30.4)	0.703	1497 (25.7)	916 (29)	0.001	2426 (27.5)	1310 (29.41)	0.021
Noninvasive ventilation, *n* (%)	104 (3.47)	28 (2.16)	0.023	154 (2.64)	81 (2.56)	0.821	258 (2.92)	109 (2.45)	0.113
Invasive ventilation, *n* (%)	111 (3.7)	24 (1.85)	0.001	140 (2.4)	41 (1.3)	<0.001	251 (2.84)	65 (1.46)	<0.001
Computed tomography of the chest, *n* (%)	466 (15.54)	183 (14.12)	0.232	570 (9.79)	253 (8.01)	0.005	1036 (11.74)	436 (9.79)	0.001
Respiratory function tests, *n* (%)	97 (3.24)	38 (2.93)	0.601	60 (1.03)	36 (1.14)	0.630	157 (1.78)	74 (1.66)	0.622
Bronchoscopy, *n* (%)	140 (4.67)	36 (2.78)	0.004	98 (1.68)	24 (0.76)	<0.001	238 (2.7)	60 (1.35)	<0.001
Lung scintigraphy, *n* (%)	25 (0.83)	6 (0.46)	0.188	2 (0.03)	2 (0.06)	0.534	27 (0.31)	8 (0.18)	0.180
Lung transplant, *n* (%)	154 (5.14)	29 (2.24)	<0.001	8 (0.14)	2 (0.06)	0.315	162 (1.84)	31 (0.7)	<0.001
Length of stay, median (IQR)	7 (8)	7 (7)	0.066	7 (7)	7 (7)	0.670	7 (7)	7 (7)	0.150
In-hospital mortality, *n* (%)	539 (17.98)	183 (14.12)	0.002	914 (15.69)	421 (13.33)	0.003	1453 (16.47)	604 (13.56)	<0.001

CCI: Charlson Comorbidity Index. AIDS/HIV: Acquired immunodeficiency syndrome/Human immunodeficiency virus. *p* value for the comparison between men and women.

**Table 3:** Prevalence of clinical conditions, procedures and in-hospital outcomes in patients hospitalized with idiopathic pulmonary fibrosis (IPF) in Spain, according to sex and diagnosis position after propensity score matching.

	Primary			Secondary			Both		
	Men	Women	*p*-Value	Men	Women	*p*-Value	Men	Women	*p*-Value
Age, mean (SD)	74.28 (10.27)	74.7 (11.22)	0.317	78.74 (9.17)	78.91 (10.44)	0.497	77.4 (9.73)	77.68 (10.84)	0.187
CCI, mean (SD)	0.83 (0.79)	0.94 (0.92)	0.015	1.57 (1.47)	1.67 (1.57)	0.013	1.35 (1.28)	1.46 (1.41)	0.001
Myocardial infarction, *n* (%)	32 (2.38)	9 (0.69)	<0.001	105 (3.37)	135 (4.27)	0.063	137 (3.08)	144 (3.23)	0.671
Congestive heart failure, *n* (%)	217 (16.16)	190 (14.66)	0.287	834 (26.8)	962 (30.45)	0.001	1051 (23.59)	1152 (25.86)	0.013
Peripheral vascular disease, *n* (%)	13 (0.97)	11 (0.85)	0.747	86 (2.76)	94 (2.98)	0.615	99 (2.22)	105 (2.36)	0.671
Cerebrovascular disease, *n* (%)	25 (1.86)	33 (2.55)	0.230	173 (5.56)	191 (6.05)	0.409	198 (4.44)	224 (5.03)	0.195
Dementia, *n* (%)	22 (1.64)	32 (2.47)	0.132	176 (5.66)	217 (6.87)	0.047	198 (4.44)	249 (5.59)	0.013
Chronic pulmonary disease, *n* (%)	223 (16.6)	237 (18.29)	0.255	703 (22.59)	670 (21.21)	0.186	926 (20.79)	907 (20.36)	0.619
Rheumatoid disease, *n* (%)	44 (3.28)	50 (3.86)	0.420	153 (4.92)	261 (8.26)	<0.001	197 (4.42)	311 (6.98)	<0.001
Peptic ulcer disease, *n* (%)	3 (0.22)	5 (0.39)	0.448	14 (0.45)	13 (0.41)	0.817	17 (0.38)	18 (0.4)	0.866
Mild liver disease, *n* (%)	53 (3.95)	54 (4.17)	0.774	124 (3.98)	123 (3.89)	0.853	177 (3.97)	177 (3.97)	0.999
Diabetes, *n* (%)	229 (17.05)	242 (18.67)	0.277	695 (22.33)	731 (23.14)	0.446	924 (20.74)	973 (21.84)	0.205
Diabetes with complications, *n* (%)	6 (0.45)	15 (1.16)	0.040	55 (1.77)	66 (2.09)	0.354	61 (1.37)	81 (1.82)	0.091
Hemiplegia or paraplegia, *n* (%)	4 (0.3)	1 (0.08)	0.192	17 (0.55)	22 (0.7)	0.450	21 (0.47)	23 (0.52)	0.762
Renal disease, *n* (%)	81 (6.03)	111 (8.56)	0.012	525 (16.87)	550 (17.41)	0.570	606 (13.6)	661 (14.84)	0.095
Cancer, *n* (%)	17 (1.27)	21 (1.62)	0.445	137 (4.4)	125 (3.96)	0.378	154 (3.46)	146 (3.28)	0.638
Metastatic solid tumor, *n* (%)	5 (0.37)	6 (0.46)	0.718	49 (1.57)	44 (1.39)	0.552	54 (1.21)	50 (1.12)	0.693
Moderate or severe liver disease, *n* (%)	4 (0.3)	9 (0.69)	0.146	20 (0.64)	26 (0.82)	0.403	24 (0.54)	35 (0.79)	0.151
AIDS/HIV, *n* (%)	0 (0)	0 (0)	NA	0 (0)	1 (0.03)	0.321	0 (0)	1 (0.02)	0.317
Pulmonary embolism, *n* (%)	7 (0.52)	10 (0.77)	0.422	70 (2.25)	43 (1.36)	0.008	77 (1.73)	53 (1.19)	0.034
Pulmonary hypertension, *n* (%)	169 (12.58)	179 (13.81)	0.351	339 (10.89)	424 (13.42)	0.002	508 (11.4)	603 (13.54)	0.002
Pneumonia, *n* (%)	30 (2.23)	48 (3.7)	0.026	497 (15.97)	380 (12.03)	<0.001	527 (11.83)	428 (9.61)	0.001
Tobacco use, *n*%	640 (47.65)	151 (11.65)	<0.001	1415 (45.47)	288 (9.12)	<0.001	2055 (46.13)	439 (9.85)	<0.001
Oxygen prior to hospitalization, *n* (%)	391 (29.11)	394 (30.4)	0.470	792 (25.45)	916 (29)	0.002	1183 (26.55)	1310 (29.41)	0.003
Noninvasive ventilation, *n* (%)	30 (2.23)	28 (2.16)	0.898	71 (2.28)	81 (2.56)	0.467	101 (2.27)	109 (2.45)	0.576
Invasive ventilation, *n* (%)	43 (3.2)	24 (1.85)	0.028	67 (2.15)	41 (1.3)	0.009	110 (2.47)	65 (1.46)	0.001
Computed tomography of the chest, *n* (%)	216 (16.08)	183 (14.12)	0.159	258 (8.29)	253 (8.01)	0.684	474 (10.64)	436 (9.79)	0.184
Respiratory function tests, *n* (%)	37 (2.76)	38 (2.93)	0.784	29 (0.93)	36 (1.14)	0.417	66 (1.48)	74 (1.66)	0.496
Bronchoscopy, *n* (%)	49 (3.65)	36 (2.78)	0.205	35 (1.12)	24 (0.76)	0.134	84 (1.89)	60 (1.35)	0.044
Lung scintigraphy, *n* (%)	9 (0.67)	6 (0.46)	0.479	1 (0.03)	2 (0.06)	0.572	10 (0.22)	8 (0.18)	0.637
Lung transplant, *n* (%)	59 (4.39)	29 (2.24)	0.002	3 (0.1)	2 (0.06)	0.643	62 (1.39)	31 (0.7)	0.001
Length of stay, median (IQR)	7 (8)	7 (7)	0.081	7 (7)	7 (7)	0.757	7 (7)	7 (7)	0.495
In-hospital mortality, *n* (%)	248 (18.47)	183 (14.12)	0.003	509 (16.36)	421 (13.33)	0.001	757 (16.99)	604 (13.56)	<0.001

CCI: Charlson Comorbidity Index. AIDS/HIV: Acquired immunodeficiency syndrome/Human immunodeficiency virus. *p* value for the comparison between men and women.

**Table 4:** Logistic regression analysis to assess variables associated to in-hospital mortality in patients with idiopathic pulmonary fibrosis (IPF) as primary diagnosis in Spain, according to sex.

	Primary	
	Men	Women
Age 18–59 years	1.58 (1.01–2.47)	3.58 (1.18–10.92)
Age 60–69 years	2.62 (1.69–4.05)	6.26 (2.1–18.72)
Age 70–79 years	3.29 (2.1–5.15)	7.28 (2.46–21.55)
Myocardial infarction	NS	NS
Congestive heart failure	NS	1.2 (1.01–1.43)
Chronic pulmonary disease	NS	NS
Diabetes	NS	NS
Hemiplegia or paraplegia	NS	NS
Cancer	3.73 (1.75–7.93)	2.57 (1.37–4.83)
Pulmonary hypertension	NS	NS
Pneumonia	2.44 (1.52–3.91)	5.98 (2.7–13.23)
Oxygen prior to hospitalization	1.37 (1.12–1.68)	2.11 (1.05–4.23)
Noninvasive ventilation	4.36 (2.82–6.73)	3.19 (1.04–9.73)
Invasive ventilation	5.55 (3.24–9.51)	4.36 (2.19–10.68)
Bronchoscopy	0.42 (0.22–0.82)	NS
Lung transplant	0.46 (0.24–0.91)	NS

NS Not significant.

## Data Availability

According to the contract signed with the Spanish Ministry of Health and Social Services, which provided access to the databases from the Spanish National Hospital Database (Conjunto Mínimo Basico de Datos; CMBD), we cannot share the databases with any other investigator, and we have to destroy the databases once the investigation has concluded. Consequently, we cannot upload the databases to any public repository. However, any investigator can apply for access to the databases by filling out the questionnaire available at http://www.msssi.gob.es/estadEstudios/estadisticas/estadisticas/estMinisterio/SolicitudCMBDdocs/Formulario_Peticion_Datos_CMBD.pdf. All other relevant data are included in the paper.

## Supplementary Materials

The following are available online at https://www.mdpi.com/article/10.3390/jcm10163474/s1, Table S1. International Classification of Disease 10th edition (ICD-10) codes for the clinical diagnosis and procedures used in this investigation. Table S2. Logistic regression analysis to assess comorbid conditions associated to in-hospital mortality in patients with idiopathic pulmonary fibrosis (IPF) in Spain, according to diagnosis position. Table S3. Sensitivity analysis. Incidence and in hospital mortality of hospital admissions with idiopathic pulmonary fibrosis (IPF), excluding all patients with a diagnosis code for any rheumatoid disease in Spain from 2016 to 2019, according to diagnosis position, sex, year and age groups. Table S4. Sensitivity analysis. Logistic regression analysis to assess variables associated to in-hospital mortality in patients with idiopathic pulmonary fibrosis (IPF) as primary diagnosis excluding all patients with a diagnosis code for any rheumatoid disease in Spain, according to sex.
