# Supplementary material for: Sex Differences in the Incidence and Outcomes of Patients Hospitalized by Idiopathic Pulmonary Fibrosis (IPF) in Spain from 2016 to 2019
Source: J Clin Med. 2021 Aug 6;10(16):3474. doi: 10.3390/jcm10163474 (PMC8396834; doi:10.3390/jcm10163474)
Supplement: Supplementary file 1 [file jcm-10-03474-s001.zip › jcm-1289243-supplementary.pdf]

Table S1. International Classification of Disease 10th edition (ICD-10) codes for the clinical diagnosis and procedures used in this investigation.

**Clinical diagnosis and procedures**	**ICD-10 codes**
Pulmonary embolism	I26.92, I26.99
Pulmonary hypertension	I27.2
Pneumonia	J09.X1, J10.00 J10.01, J10.08, J11.0, J11.00, J11.08<br>All from J12 to J18
Oxygen prior to hospitalization	Z99.81
Tobacco use	Z72.0, F17.2--, Z87.891
Noninvasive ventilation	5A09357, 5A09457, 5A09557
Invasive ventilation	5A1945Z, 5A1955Z, 5A1935Z
Computed tomography of the chest	BP2WYZZ, BP2W0ZZ, BP2W1ZZ, BW24YZZ, BW24Y0Z, BW24ZZZ, BW240ZZ, BW2400Z, BW241ZZ, BW2410Z
Respiratory function tests	4A09X1Z, 4A0971Z, 4A0981Z, 4A19X1Z, 4A1971Z
Bronchoscopy	0BJK8ZZ, 0BJL8ZZ, 0BJ08ZZ, 0BBK8ZX, 0BBL8ZX, 0BBM8ZX, 0B9K8ZX, 0B9L8ZX, 0B9M8ZX
Lung scintigraphy	CB1YYZZ, CB12TZZ, CB12VZZ, CB2YYZZ, CB3YYZZ
Lung transplant	0BYK0Z0, 0BYK0Z1, 0BYK0Z2, 0BYL0Z0, 0BYL0Z1, 0BYL0Z2, 0BYM0Z0, 0BYM0Z1,0BYM0Z2

Table S2. Logistic regression analysis to assess the effect of sex and comorbid conditions on the in-hospital mortality in patients with idiopathic pulmonary fibrosis (IPF) as primary diagnosis in Spain.

	**PRIMARY**
Age 18-59 years	2.87(1.38-5.95)
Age 60-69 years	6.15(3.01-12.55)
Age 70-79 years	7.09(3.48-14.44)
Myocardial infarction	1.46(1.04-2.04)
Congestive heart failure	NS
Diabetes	0.66(0.48-0.9)
Hemiplegia or paraplegia	NS
Cancer	4.35(1.2-15.82)
Pneumonia	2.46(1.47-4.11)
Oxygen prior to hospitalization	1.41(1.12-1.77)
Noninvasive ventilation	4.39(2.51-7.67)
Invasive ventilation	5.34(2.94-9.69)
Bronchoscopy	0.49(0.31-0.82)
Lung transplant	0.54(0.34-0.97)
Men	1.43(1.15-1.77)

NS Not significant

Table S3. Sensitivity analysis. Incidence and in hospital mortality of hospital admissions with idiopathic pulmonary fibrosis (IPF), excluding all patients with a diagnosis code for any rheumatoid disease in Spain from 2016 to 2019, according to diagnosis position, sex, year and age groups.

	PRIMARY			SECONDARY			BOTH		
	Men	Women	P-value	Men	Women	P-value	Men	Women	P-value
Incidence (2016-2019), n(Inc/105)	2947(3.96)	1246(1.58)	<0.001	5638(7.56)	2898(3.67)	<0.001	8585(11.53)	4144(5.24)	<0.001
2016, n(%)	698(23.69)	352(28.25)	0.002	1286(22.81)	774(26.71)	<0.001	1984(23.11)	1126(27.17)	<0.001
2017, n(%)	796(27.01)	355(28.49)	0.326	1533(27.19)	855(29.5)	0.024	2329(27.13)	1210(29.2)	0.015
2018, n(%)	670(22.73)	272(21.83)	0.521	1419(25.17)	617(21.29)	<0.001	2089(24.33)	889(21.45)	0.001
2019, n(%)	783(26.57)	267(21.43)	<0.001	1400(24.83)	652(22.5)	0.017	2183(25.43)	919(22.18)	<0.001
Age 18-59 years, n(%)	349(11.84)	111(8.91)	0.005	352(6.24)	123(4.24)	0.035	701(8.17)	234(5.65)	0.001
Age 60-69 years, n(%)	825(27.99)	257(20.63)	<0.001	954(16.92)	294(10.14)	<0.001	1779(20.72)	551(13.3)	<0.001
Age 70-79 years, n(%)	1090(36.99)	375(30.1)	<0.001	2003(35.53)	807(27.85)	<0.001	3093(36.03)	1182(28.52)	<0.001
Age 80 year or over, n(%)	683(23.18)	503(40.37)	<0.001	2329(41.31)	1674(57.76)	<0.001	3012(35.08)	2177(52.53)	<0.001
In hospital mortality, n(%)	528(17.92)	177(14.21)	0.003	902(16)	398(13.73)	0.006	1430(16.66)	575(13.88)	<0.001

Inc/105: Incidence per 100,000 people. P value for comparison of men versus women.

Table S4. Sensitivity analysis. Logistic regression analysis to assess variables associated to in-hospital mortality in patients with idiopathic pulmonary fibrosis (IPF) as primary diagnosis excluding all patients with a diagnosis code for any rheumatoid disease in Spain, according to sex.

	PRIMARY	
	MEN	WOMEN
Age 18-59 years	1.7(1.09-2.65)	3.95(1.07-16.31)
Age 60-69 years	2.77(1.79-4.28)	7.85(1.26-22.97)
Age 70-79 years	3.31(2.11-5.19)	9.57(1.67-30.1)
Congestive heart failure	NS	1.47(1.02-2.28)
Cancer	3.59(1.68-7.65)	3.01(1.19-8.62)
Pneumonia	2.4(1.49-3.86)	3.87(1.91-14.85)
Oxygen prior to hospitalization	1.42(1.15-1.74)	1.55(1.1-2.19)
Noninvasive ventilation	4.22(2.74-6.49)	5.28(2.24-12.46)
Invasive ventilation	5.69(3.3-9.79)	4.83(1.22-19.13)
Bronchoscopy	0.41(0.21-0.79)	NS
Lung transplant	0.43(0.22-0.84)	NS

NS. Not significant
